# Assessment of viral RNA in idiopathic pulmonary fibrosis using RNA-seq

**DOI:** 10.1186/s12890-020-1114-1

**Published:** 2020-04-03

**Authors:** Qinyan Yin, Michael J. Strong, Yan Zhuang, Erik K. Flemington, Naftali Kaminski, Joao A. de Andrade, Joseph A. Lasky

**Affiliations:** 1 Section of Pulmonary Diseases, Critical Care and Environmental Medicine, Department of Medicine, Tulane University School of Medicine, 1430 Tulane Avenue, New Orleans, LA 70112 USA; 2 Department of Pathology and Laboratory Medicine, Tulane University School of Medicine, 1430 Tulane Avenue, New Orleans, LA 70112 USA; 3 Section of Pulmonary, Critical Care and Sleep Medicine, Yale University, 300 Cedar Street, Ste S441D, New Haven, CT 06519 USA; 4 Division of Allergy, Pulmonary, Critical Care Medicine, Department of Medicine, Vanderbilt University, 1161 21st Avenue South, B1317 MCN, Nashville, TN 37232-2650 USA

**Keywords:** Idiopathic pulmonary fibrosis, IPF, RNA-seq, Viruses, EBV, HCV, HERV-K, Herpesvirus saimiri

## Abstract

**Background:**

Numerous publications suggest an association between herpes virus infection and idiopathic pulmonary fibrosis (IPF). These reports have employed immunohistochemistry, in situ hybridization and/or PCR, which are susceptible to specificity artifacts.

**Methods:**

We investigated the possible association between IPF and viral RNA expression using next-generation sequencing, which has the potential to provide a high degree of both sensitivity and specificity. We quantified viral RNA expression for 740 viruses in 28 IPF patient lung biopsy samples and 20 controls. Key RNA-seq results were confirmed using Real-time RT-PCR for select viruses (EBV, HCV, herpesvirus saimiri and HERV-K).

**Results:**

We identified sporadic low-level evidence of viral infections in our lung tissue specimens, but did not find a statistical difference for expression of any virus, including EBV, herpesvirus saimiri and HERV-K, between IPF and control lungs.

**Conclusions:**

To the best of our knowledge, this is the first publication that employs RNA-seq to assess whether viral infections are linked to the pathogenesis of IPF. Our results do not address the role of viral infection in acute exacerbations of IPF, however, this analysis patently did not support an association between herpes virus detection and IPF.

## Background

Idiopathic pulmonary fibrosis (IPF) is a progressive disease with insidious onset in older people that progresses relentlessly in the absence of therapy to disability and death [1]. Although multiple risk factors, including viral infection, have been linked to IPF, studies are inconsistent and its etiology remains unclear. To date, more than 14 viruses have been investigated for a potential role in initiation and progression of IPF, including RNA viruses such as Hepatitis C (HCV), and DNA viruses such as human herpes viruses (HHVs), adenoviruses, human endogenous retrovirus E (HERV-E), transfusion transmitted virus (TTV) and parvovirus B19 [2–7]. However, the studies on the relationship between virus expression and development of IPF are conflicting. While some studies show that virus infection is associated with IPF, other manuscripts show no viral association with IPF [8–11], which may be due to the differences in virus distribution and sensitivity and/or specificity of the techniques employed between studies.

Given the fact that alveolar epithelial cells are abnormal and likely contribute to the pathobiology of IPF, we believe that epithelial perturbation may contribute to inducing and maintaining IPF. Herpes viruses can infect many different cell types, including epithelial cells with two infection stages, the lytic stage and the latent stage. Both lytic genes and latent genes could interact with cellular genes to contribute to IPF initiation, progression and/or maintenance. Viral genes may regulate cellular gene expression and induce fibrogenesis. For example, Type I collagen can be induced by adenovirus [12]. HSV-1 stimulates endoplasmic reticulum (ER) stress and apoptosis [3], and these processes are implicated in the pathobiology of IPF. HCMV infection induces the unfolded protein response (UPR) and it’s related signaling pathways elF2alpha kinase PERK, and causes endoplasmic reticulum stress [13]. CMV, KSHV and EBV also induce endoplasmic reticulum stress and the UPR [14]. Murine studies demonstrate that the latent genes of MHV-68 induce lung fibrosis in mice via TGF-β, vascular endothelial growth factor, CCL2, CCL12, TNF-α and IFN-γ [15]. EBV lytic gene expression also activates TGF-β expression in alveolar epithelial cell lines [16] and in primary corneal epithelial cells [17] where EBV can induce epithelial-mesenchymal transition (EMT) [18]. Moreover, the fibrotic cytokine milieu in IPF lung may activate virus replication and further promote virus gene expression in fibrotic lung. TGF-β promotes EBV reactivation from latency to the lytic replication stage and further induces latent membrane protein expression, which synergizes with TGF-β1 to induce EMT in lung epithelial cells [19]. Given these mechanisms, it is reasonable to speculate that viruses may play a role in the pathogenesis of IPF.

If viruses are responsible for causing IPF, then viral screening or anti-viral treatment may provide a diagnostic test or a potential treatment. Previously employed techniques, including immunohistochemistry, fluorescence in situ hybridization (FISH), gene array and PCR, are not sufficiently sensitive or specific. Thus, a more sensitive and reliable technique is required to qualify and quantity viruses at the DNA, or RNA levels. Next-generation sequencing offers high sensitivity, specificity and reproducibility in the detection of low levels of gene expression as well as a broad dynamic range afforded by the high sequencing depth. New high throughput technologies have been used to generate comprehensive sequencing data for the identification and quantification of known and novel genes in several diseases [20]. Moreover, RNA-seq has the potential to further elucidate the mechanisms of pathogenesis of IPF by identifying novel viruses not previously implicated by PCR or array based methods. Here we have utilized RNA-seq for the detection of virus expression in lung tissue from patients with IPF and their age-matched controls.

## Methods

### Sample description

Lung tissue samples for the first group were obtained from remnants of surgical biopsies from the University of Alabama at Birmingham (UAB) according to IRB (approval number N120410001 and 12-334398E). They included 5 control lungs and 12 IPF lungs. The control lung samples were obtained from histological disease-free margins from patients undergoing resection of lung adenocarcinoma. The diagnosis of IPF patients was made by clinicians, pathologists and radiologists according to diagnostic criteria of the American Thoracic Society and the European Respiratory Society [21]. The detailed demographics for the first group are available at GEO along with raw RNA-seq data (accession numbers: GSE138239 for poly(A) selected RNA-seq data and GSE138283 for non-poly(A) selected RNA-seq data). The demographics and description for lung tissue samples for the second and third groups were previously published [22–24]. IPF patients for the second group of samples (Pittsburgh) and the third group of samples (FFPE) were vetted using the 2000 and 2011 ATS/ERS guidelines respectively [21, 25].

### RNA-sequencing data acquisition from IPF and control lung

For this report, we analyzed RNA expression from 3 groups that in total were derived from 28 IPF and 20 control lung specimens. The first group included samples from 12 IPF patients and 5 controls, and encompassed patients who underwent lung biopsies at the University of Alabama at Birmingham. Total RNA was prepared using Qiagen’s RNeasy kit (cat#74104). Poly(A) selected RNA-sequencing (RNA-seq) was performed at UAB for the first group (one control (3007) and one IPF (2053) sample was not analyzed by RNA-seq because the aliquot of RNA was considered to be of insufficient quality for these two samples). We repeated RNA-sequencing on this same group at Tulane, but we did not select for polyadenylation because some viral genes are not polyadenylated. Non-poly(A) selected RNA-seq was performed using the Illumina NextSeq 550 located within the NextGen Sequencing Core at the Tulane Center for Translational Research in Infection & Inflammation. Ribosomal RNA was removed from 1 μg total RNA for both poly(A) selected and non-poly(A) selected RNA-seq, and a library was prepared using TruSeq stranded mRNA (polyA+) for poly(A) selected RNA-seq or using TruSeq stranded total RNA ribozero [12] for non-poly(A) selected RNA-seq from Illumina. The second group, including 10 IPF samples and 10 age-matched controls, were from a poly(A) selected RNA-seq dataset provided from the University of Pittsburg. The lung tissue for the Pittsburg group was part of the LTRC (Lung Tissue Research Consortium) specimen bank that was funded by NHLBI biospecimen repository. The third group, including 6 IPF lungs and 5 controls, was obtained from a RNA-seq dataset downloaded from the sequence read archive (SRA, PRJNA326784, https://www.ncbi.nlm.nih.gov/sra?linkname=bioproject_sra_all&from_uid=326784), and was generated with non-poly(A) selected total RNA [24]. RNA from the third group was isolated from paraffin-embedded tissue. Although we acknowledge that fixation in group 3 had the potential to damage nucleic acids, RNA-seq for these samples was considered reliable based on the original report (~ 62 million mapped reads in 116 million reads at 50 bp per sample) [24].

### Virome analysis of the RNA-sequencing data

Raw RNA-seq data was aligned to a genome reference containing the human genome (hg19; genome reference consortium GRCh37) plus a library of 740 known mammalian viral sequences that have been documented by the NCBI (National Center for Biotechnology Information). Alignments were performed using the transcript aligner STAR (Spliced Transcripts Alignment to a Reference) version 2.3.0 and version 2.5.2a. Uniquely mapped viral and human reads were quantified using in-house computational pipelines. The first script extracts all the reads mapping to virus sequences and writes the output to one file for each sample. The second script takes as input all the viral sequence information from the first script and removes any duplicate reads. The output is a list of all the uniquely mapped viral sequences for each sample. The third script takes as input the uniquely mapped reads for each sample and counts the number of reads mapping to each virus in each sample. The output is a compiled file that contains the virus chromosome name followed by the number of occurrences in each aligned file. As a complementary approach, we also analyzed mapped reads using the metatranscriptomics pipeline, RNA CoMPASS for entire metatranscriptome analysis [26].

### cDNA synthesis and RT-PCR

cDNA was synthesized with 1μg RNA following the manufacturer’s instructions within the Bio-Rad iScrip^tm^ cDNA Synthesis Kit (cat#170–8891). PCR was performed with 2 μl of 10X diluted cDNA in a 20 μl volume according to the manufacturer’s protocol (BioRAD cat#170–8880). PCR conditions: for EBV we used 3 min at 95 °C, 40 cycles of 15 s at 95 °C and 30 s at 60 °C then 40 s at 72 °C [27]; to detect HCV, we followed the methods of Lin et al. [28]; to detect HHV-7, we followed Caserta’s method [29]; to detect saimiri expression, we followed Folcik ‘s method [30] using the primers listed on Table 1. HERV-K strand-specific nested-RT-PCR products from primers designed to detect RNAs spliced at the conventional envelope (env) mRNA splice junction (sense strand, 1 × −env) following the nested-PCR and quantitative RT-PCR protocol of Agoni et al. [32]. In brief, 1x-env products were amplified with cDNA reverse transcript using primers for RT-env-1-Rev then nested-PCR with primers env (1) & [31]. The primer sequences used for RT-PCR analysis were listed in Table 1. Relative transcript expression levels were calculated using the ∆∆Ct method and the fold change of relative transcript expression was calculated by ∆∆Ct of IPF/ ∆∆Ct of control (CNTL).

**Table 1:** Nucleotide sequence of primer sets used for RT-PCR analysis in this study

Genes		Forward nucleotide sequence (5′ → 3′)	Revers nucleotide sequence (5′ → 3′)
EBV EBERs		GACTCTGCTTTCTGCCGTCT	AATAGCGGACAAGCCGAATA
HERV-K	RT-env-1-Rev		CACCGCACTATTGGCCACA
	Nested-PCR env(1)	AGGGAAAAACCGCCTTAGGG	CACCGCACTATTGGCCACA
	Nested-PCR env [31]	TGCGGGCAGCAATACTGCT	CGCACTATTGGCCACACATTC
	Quantitative PCR env	TCACATGGTAAGCGGGATGTC	CGCACTATTGGCCACACATTC
	Quantitative PCR LTR	AGGGAAAAACCGCCTTAGGG	AGCAGACAAACATGTGAACAAAGG
	LTR-Fwd |-Rev	CGTGGGAAGGGAAAGACCTGA	AGCAGACAAACATGTGAACAAAGG
GAPDH	GAPDH	AGATCATCAGCAATGCCTCCT	AGTCTTCTGGGTGGCAGTG
HCV	p3804|p305	GTATCTCGAGGCGACACTCCACCATAGAT	ATACTCGAGGTGCACGGTCTACGAGACCT
	p302|p304	CCACCATAGATCTCTCCCCTGT	CACTCTCGAGCACCCTATCAGGCAGT
Saimiri virus	Human cyclin D1	CGGAGGAGAACAAACAGATCATCCGCAAAC	GTGTGAGGCGGTAGTAGGACAGGAAGTTGT
	Viral cyclin D1	ACTGCTTACCTGGATGCATCTGCTCTGTGA	GCAAGTACAGCTTCAGTGTGTCCCATTTCAGTGC
HHV-7	G1|G2	CATGCACAACGCAAGCTCTACTA	ACGTAGTTTCGTGCAGTTGTATCGT
	G3|G4	GCTTGTTAGAATACACAAGATGTACA	CTGTCTAATAATGTCTATGTCTCTCCA

### Statistical analyses

RT-PCR was performed in triplicate for each sample. In order to test the significant difference of RT-PCR data and/or RNA-seq reads (RPHM), we used student’s t-tests and F tests that were performed using GraphPad Prism. T-tests were used to compare differences between control and IPF groups, and the F tests were employed to compare variance within the groups (control group or IPF group). Differential expression analysis of RNA-Seq was carried out using the EBSeq statistical package. Scatter plots depict the mean with the standard error of the mean [33]. Statistical significance was defined at an alpha value of *p* < 0.05. Results are expressed as mean ± SEM.

## Results

### Quantitation of viral gene expression using RNA-sequencing

Although we observed that the Shamonda virus averages 10 reads per million human mapped reads (RPHM) in poly(A) selected RNA-seq, this is likely an artifact and not true infection because viral reads were detected in every sample analyzed, including the normal controls. In addition, manual BLAST showed that the actual reads hit to human sequences that were mistakenly being called Shamonda virus, and a few repeat reads have been clonally amplified and resulting in such a high read number. The next most commonly detected virus was human adenovirus C with 1 read per million human mapped reads (Table 2). The mapped reads of other viruses were very low (under 1 RPHM, Table 2) in poly(A) selected RNA-seq. This could be due to exclusion of viral RNA that is not polyadenylated. To detect viral encoded non-coding RNAs, we performed a non-poly(A) RNA-seq using ribodepleted RNA libraries for our initial group 1 samples (5 controls and 12 IPF lungs). Non-poly(A) selected RNA-seq detected more virus than poly(A) selected RNA-seq, including tick-born encephalitis virus, herpesvirus 2 (HHV-2, HSV-2), Roseolovirus (HHV-6B) and EBV (HHV-4, Table 3, Table S3). However, there were no significant differences between control and IPF (Table 3, Tables S3, S4). These data were confirmed by analysis of viral RNA expression using another non-poly(A) selected RNA-seq datasets (the third group dataset, Table S5). Overall, none of the samples from either the control or IPF groups reached a virus detection threshold high enough to qualify as positive. We conclude that there are no viruses associated with IPF tissue samples (Table 2, Table S1).

**Table 2:** Summary of the number of virus mapped reads per million human mapped reads (RPHM) for IPF and control lung specimens from first and second group in poly(A) selected RNA-seq and third group in non-poly(A) selected RNA-seq. Viruses were displayed here if at least one viral mapped read was detected in at least one sample. The total mapped reads of human are for quality control

Virus name	Control				IPF			
	# of samples	% of samples	Min	Max	# of samples	% of samples	Min	Max
1st group								
Human	4	100%	26,511,620	39,945,034	11	100%	23,500,148	44,071,337
Cytomegalovirus	0	0%	0	0	1	9%	0.80	0.80
Adenovirus C	0	0%	0	0	2	18%	0.03	0.07
Mouse mammary tumor virus	1	25%	0.03	0.03	4	36%	0.03	0.05
Simbu virus	3	75%	0.03	0.23	5	45%	0.03	0.20
Immunodeficiency virus 1	0	0%	0	0	1	9%	0.03	0.03
Hepatitis C virus genotype 1	0	0%	0	0	1	9%	0.03	0.03
Hepatitis C virus genotype 2	0	0%	0	0	1	9%	0.04	0.04
Papillomavirus 110	0	0%	0	0	1	9%	0.02	0.02
Shamonda virus	4	100%	5.407	15.489	11	100%	0.03	15.98
2nd group								
Human	10	100%	19,847,360	37,420,687	10	100%	21,826,038	41,977,536
Cytomegalovirus	0	0%	0.00	0.00	2	20%	0.07	0.14
Herpesvirus 6A	0	0%	0.00	0.00	1	10%	0.04	0.04
Herpesvirus 7	2	20%	0.03	0.04	0	0%	0	0
Adenovirus C	10	100%	0.05	0.35	10	100%	0.05	1.05
Simian virus 40	1	10%	0.03	0.03	0	0%	0	0
Papillomavirus 16	1	10%	0.04	0.04	0	0%	0	0
Shamonda virus	10	100%	0.35	6.28	10	100%	1.42	11.17
3rd group								
Tick-borne encephalitis virus	2	40%	0.00	0.11	2	33%	0.00	0.06
Hepatitis C virus genotype 1	5	100%	0.03	3.63	6	100%	0.14	1.07
Hepatitis C virus genotype 6	3	60%	0.00	0.04	2	33%	0.00	0.03
Hepatitis C virus genotype 2	4	80%	0.00	0.11	5	83%	0.00	0.09
Adenovirus C	5	100%	0.02	0.33	6	100%	0.78	3.27
Adenovirus E	0	0%	0.00	0.00	1	17%	0.00	0.03
Herpesvirus 1	0	0%	0.00	0.35	1	17%	0.00	0.06
Herpesvirus 2	1	20%	0.00	0.04	5	83%	0.00	0.03
Epstein-Bar virus	1	20%	0.00	0.04	1	17%	0.00	0.14
Cytomegalovirus	4	80%	0.00	0.41	2	33%	0.00	0.09
Herpesvirus 7	3	60%	0.00	0.07	0	0%	0.00	0.00
Kaposi’s sarcoma-associated herpesvirus	1	20%	0.00	0.02	1	17%	0.00	0.02
Cutthroat trout virus	0	0%	0.00	0.00	3	50%	0.00	0.03
Measles virus	0	0%	0.00	0.00	2	33%	0.00	0.03
Abelson murine leukemia virus	5	100%	0.01	0.31	1	17%	0.00	0.03
Shamonda virus	3	60%	0.00	0.05	1	17%	0.00	0.03
Simian virus 40	5	100%	0.02	0.46	5	83%	0.00	0.06
Papillomavirus 28	0	0%	0.00	0.00	1	17%	0.00	0.03
Papillomavirus 100	0	0%	0.00	0.00	1	17%	0.00	0.03

**Table 3:** Comparison of the virome reads between non-poly(A) selected RNA-seq (non-poly(A)) with poly(A) selected RNA-seq (poly(A)) from first group of lung tissue. The numbers correspond to the average virome reads per million human mapped reads

Viruses	Non-poly(A)		Poly(A)	
	Controls	IPF	Controls	IPF
Cytomegalovirus	0.15	0.18	0.00	0.80
Hepatitis C virus genotype 2	2.05	2.57	0.00	0.04
Hepatitis C virus genotype 6	0.49	0.61	0.00	0.00
Hepatitis C virus genotype 1	0.15	0.16	0.00	0.03
Tick-borne encephalitis virus	0.23	0.22	0.00	0.00
Abelson murine leukemia virus	0.09	0.14	0.06	0.05
Shamonda virus	0.08	0.09	9.15	3.52
Cutthroat trout virus	0.02	0.02	0.00	0.00
Murine type C retrovirus	0.02	0.01	0.00	0.00
Simbu virus	0.02	0.02	0.10	0.08
Moloney murine leukemia virus	0.02	0.00	0.03	0.02
Herpesvirus 2	0.01	0.01	0.00	0.00
Epstein-barr virus	0.01	0.01	0.00	0.00
Herpesvirus 6B	0.01	0.00	0.00	0.00
Adenovirus C	0.00	0.00	0.00	0.05
Immunodeficiency virus 1	0.00	0.00	0.00	0.03
Papillomavirus 110	0.00	0.00	0.00	0.02
Mouse mammary tumor virus	0.00	0.00	0.03	0.03
HERV-K	96.35	94.04	29.36	52.11
HERV-W	4.58	4.00	0.21	0.44
HERVs	102.80	99.41	32.87	54.39

### Screening for EBV, HCV, HHV-7 and herpesvirus saimiri RNA using real-time RT-qPCR

**Fig. 1:**
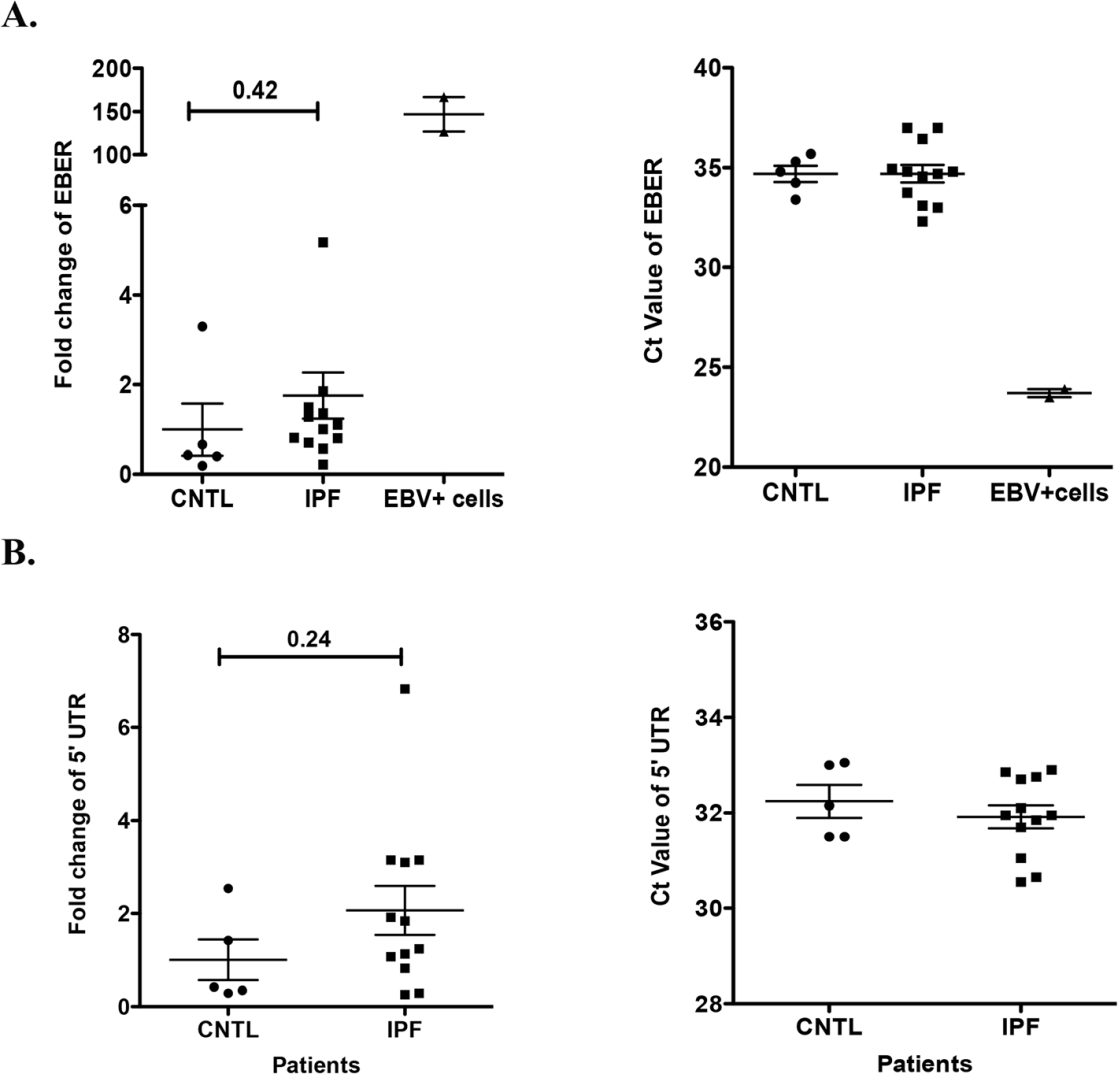
Evaluation of EBV and HCV expression levels in IPF and control lung specimens by RT-qPCR. **a** To evaluate EBV expression levels, RT-qPCR was performed using primers against EBER. **b** HCV expression was assessed using primers against the 5’UTR

To confirm our RNA-seq results, we performed serial RT-qPCR on the first group of specimens (12 IPF and 5 control lung RNAs). This was not performed on the second and third group because we only had the data sets and not the RNA. EBV has two major infection gene expression programs, the latency associated gene expression program and the lytic gene expression program, which are uniquely utilized depending on cell type. Since it is not known which cell type might harbor EBV within IPF lung, and to avoid “lack of detection” errors due to EBV infection status, primers spanning the EBV latent genes, EBNA1, Qp and LMP1, as well as the EBV lytic gene Zta were employed for RT-qPCR. No EBV latent or lytic gene expression was detected using RT-qPCR, suggesting that neither the latent nor the lytic forms of EBV were present in the lungs of IPF patients or the control group (data not shown). However, using primers that span the EBV-encoded noncoding small RNAs, EBER1 to EBER2, we detected a very low level of EBERs expression in both the IPF and control specimens, with cycle threshold [34] values over 33 cycles and with no significant difference between the two groups (Fig. 1a). This data is consistent with the analysis of the non-polyA selected RNA-seq.

Other ubiquitous herpes viruses have also been reported to be associated with IPF, including herpes simplex virus type 1 (HSV-1), HHV-6, − 7 and − 8 and cytomegalovirus (CMV) [2]. Our RNA-seq data detected sporadic and very low virus mapped reads per million human mapped reads (RPHM – reads per million human mapped) for these viruses; HHV-5 with 1 RPHM in IPF lung and 2 RPHM in control lung; HHV-6 with 1 RPHM read in control and HHV-7 with 2 RPHM in IPF (Table 2). RT-qPCR Ct values for these viruses were around 40, and therefore not reliable for quantification of these HHVs (data not shown). Chronic infection of HCV has been implicated in liver fibrosis; however, it is still debatable whether HCV can cause pulmonary fibrosis. While some research indicates that HCV infection may play an important role in the pathogenesis of IPF [4, 5], others have not detected HCV RNA in IPF samples, despite detection in some specimens using ELISA [10, 35]. No HCV mapped reads were detected in any of our IPF or control lung specimens using RNA-seq (Table S1). A nested real-time RT-qPCR assay with primers spanning the 5-UTR of HCV [28, 36] detected very low levels of HCV transcripts with Ct value over 30 cycles (Fig. 1b). Importantly, the ∆∆Ct for HCV was not significantly different between IPF and controls (Fig. 1b).

**Fig. 2:**
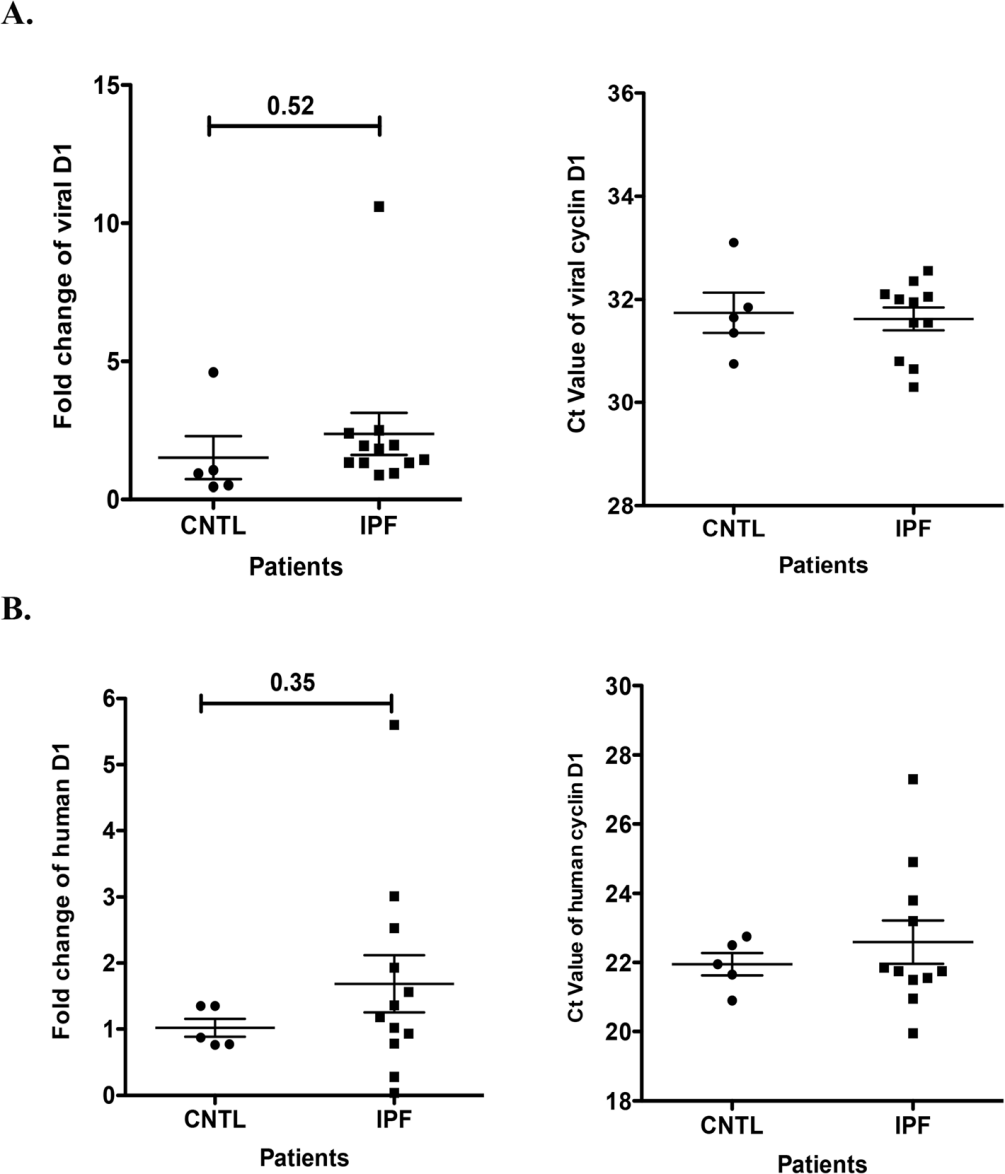
Detection of herpesvirus saimiri expression in IPF and control lung specimens. **a** Saimiri virus was assessed using RT-qPCR using primers designed for viral cyclin D1. **b** Human cyclin D1 was evaluated using RT-qPCR using primers designed for human cyclin D1

More recently, Folcik et al. reported that IPF is associated with herpesvirus saimiri but not with other herpesviruses such as EBV, KSHV, CMV or HSV I/II [30]. They detected herpesvirus saimiri DNA and RNA in all 13 IPF cases and none of their controls. Herpesvirus saimiri is a member of the rhadinovirus genus, which also includes Kaposi’s sarcoma-associated herpesvirus, and can infect humans and squirrel monkeys without causing disease. Around 4.0–7.3% of humans are seropositive and express viral proteins such as viral cyclin D [37]. Although no substantial herpesvirus saimiri virus reads were detected in any of the IPF and control specimens using RNA-seq (Table 2 & S1), we still performed RT-qPCR to assess expression of herpesvirus saimiri using primers against viral cyclin D1 and viral ORF73 (a conserved viral gene). We did not detect significant expression of ORF73 in IPF patient samples compared to controls (data not shown). We observed high expression of human cyclin D1 (Fig. 2b) and very low expression of viral cyclin D1 (Ct value over 30, Fig. 2a) in both IPF and control samples, indicating lack of an association between herpesvirus saimiri and IPF.

### HERV-K gene expression and coverage in IPF patients

**Fig. 3:**
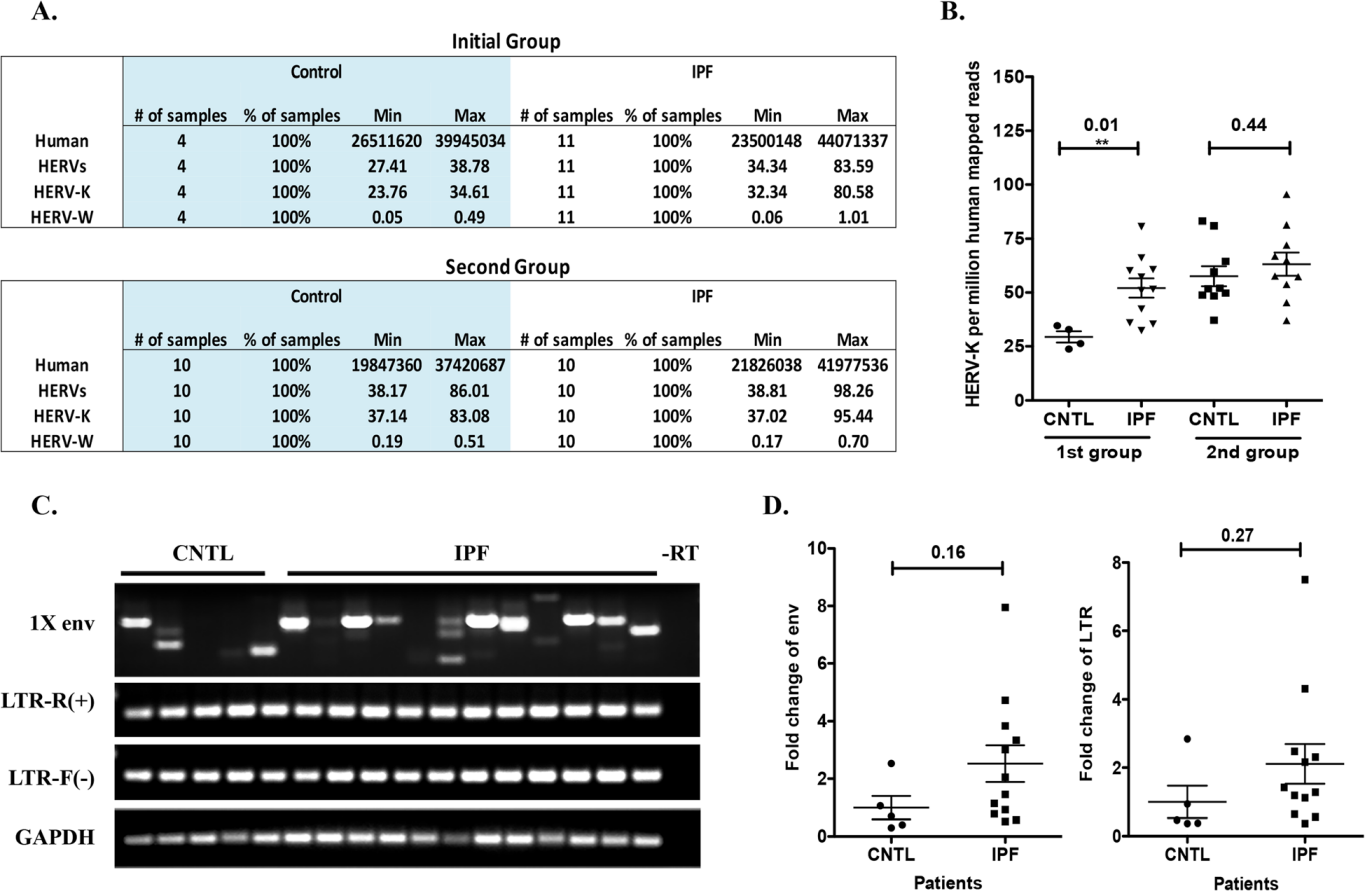
Detection of HERV-K transcripts in human lung tissue. **a** Counts for HERV mapped reads and human mapped RNA-seq reads for IPF and control RNA. **b** Quantification of HERV-K RNA-seq mapped reads. **c** Strand-specific RT-PCR was performed to detect viral env transcripts and LTR expression, and the products were resolved by electrophoresis (−RT indicates no reverse transcriptase control). **d** Detection of HERV-K gene expression by qualitative RT-PCR using primers designed for env and LTR. The relative transcript expression levels were calculated using the ∆∆Ct method and fold change was calculated by the ∆∆Ct of IPF/∆∆Ct of control (CNTL)

HERV sequences make up about 4.9% of the human genome. HERV-K research has been assessed in autoimmune disorders and oncogenesis, yet to date we are not aware of any literature to assess its possible role in pulmonary fibrosis. Recently, RT-PCR results have suggested that HERV-K env mRNA was increased in PBMC and skin biopsies of morphea/localized scleroderma [38]. This study suggests that HERV-K env may be functionally linked to fibrosis. HERV-K gene expression could theoretically promote IPF through cell stress, and HERV-K expression is reported to be higher with EBV infection [39]. Therefore, we evaluated whether HERV-K genes are upregulated in IPF lung. Notably, of the viruses analyzed in poly(A) selected RNA-seq, HERV-K was the virus with the highest read numbers (23 to 83 HERV-K mapped reads per million human mapped reads in both IPF and control samples) (Fig. 3a & Table S2). Statistical analysis showed about a 2-fold increase in the 11 IPF patient samples compared 5 controls in group 1 (Fig. 3a & b). However, no statistical difference was evident between IPF and controls in the second group (Fig. 3a & b). These data were confirmed by non-poly(A) selected RNA-seq in the initial group and the third group. Non-poly(A) selected RNA-seq detected more HERV mapped reads than poly(A) selected RNA-seq (Table 3, Table S4, Table S5). Overall we were not able to make an association between HERV-K gene expression and IPF.

Quantitative RT-PCR of the HERV-K env and long terminal repeat (LTR) regions show that the expression levels of env and LTR were higher in IPF than in controls (two-fold difference, Fig. 3d), which corroborates the RNA-seq data. Next, strand-specific nested RT-PCR was performed with primers spanning the HERV-K env and LTR regions in group 1. The primers were originally designed to detect viral 1x env splicing transcripts [32]. Since HERV-K can be transcribed from the LTR at either or both directions, the sense strand and anti-sense strand, we performed strand-specific RT-PCR to detect the plus strand and the minus strand using forward (LTR-Fwd) or reverse primers (LTR-Rev) for reverse strand transcription of the LTR. As shown in Fig. 3c, we found no statistical difference in expression of env and LTR from either direction between IPF and controls. We observed that there were several different sizes of env spliced transcripts. Eleven of 12 (91.7%) IPF samples were env positive, compared to 3 of 5 (60%) controls, and the majority of env transcripts were large in IPF (9 of 11), compared with 1 of 3 env in controls (Fig. 3c). In summary, the spliced env appears preferentially expressed in IPF, and we do not yet know whether the large env may play a role in IPF pathogenesis.

## Discussion

Here we used RNA-seq to characterize 740 virus gene expression profiles in 28 IPF biopsies and 20 age-matched controls. RNA-seq did not provide evidence for an association between any virus and IPF. Studies using RT-PCR for HERV-K, saimiri, EBV, HHV7 and HCV by RT-PCR corroborated the RNA-seq results. Our findings provide a new scope for exploring the causes of IPF by using the sensitive RNA-seq method.

To enable us to analyze both viral poly(A) and non-poly(A) mRNA in the same sample, we divided total RNA extracted from surgical lung biopsies into a poly(A) enriched fraction via oligo (dT) for poly(A)-selected RNA-seq, and a non-poly(A) fraction for non-poly(A)-selected RNA-seq. Similar to eukaryotic mRNAs, viral mRNAs have two main types: poly(A) and non-poly(A) transcripts, based on the presence or absence of a poly(A) tail at their 3′-end. Poly(A) mRNA transcripts represent the majority of viral mRNA, however some viruses express non-poly(A) mRNA including miRNA and lncRNA. Important examples are herpesvirus EBV-encoded non-coding small RNAs (EBER), and adenovirus-encoded non-coding RNA VA (viral associated) RNAs. Viral-encoded non-poly(A) RNAs have an essential role in a variety of physiological conditions and in several illnesses, including viral life cycle and function, host cell immune evasion and transformation [40]. Specifically, EBERs are highly abundant in all latently EBV-infected cells and play a significant role in the pathogenesis of EBV infection including contributions to EBV-mediated oncogenesis such as Burkitt’s lymphoma, gastric carcinoma and nasopharyngeal carcinoma through regulation of apoptosis and/or several cytokines [41]. As such, EBERs are the gold standard clinic markers for detection of EBV latent infection in specimens. For this reason, we analyzed viral poly(A) and non-poly(A) mRNAs separately on the same sample from the first group.

EBV appears to be the most commonly investigated virus in IPF. Previous research has suggested that IPF is linked to EBV, while other studies, using some common techniques such as PCR with primers from the EBV BamHI W repeats or the EBER gene, FISH with an EBER probe, and IHC with antibody against the viral capsid antigen (VCA) or the latent membrane protein 1, have found no link [9]. Here, we did not detect EBV latent or lytic gene expression differences using RNA-seq or real-time RT-qPCR between the lungs of IPF patients compared to control lungs. Nevertheless, we detected very low level of EBER in both IPF and control lung with no difference between the two groups, and this is not unexpected since most people are latently infected with EBV. Notably, EBV EBERs are the most highly expressed EBV latent genes, typically with greater than 1 million RNA molecules per cell [42]. EBERs were detected at very low-levels in the non-poly(A) selected RNA-seq dataset (Table S3) and in real-time RT-qPCR, but not in poly(A) selected RNA-seq. Their quantification failed to demonstrate enhancement of EBV gene expression in IPF specimens, thus implying that the EBV virus is not associated with IPF lung any more than with normal lung.

HERV-K expression was examined because some reports have indicated that it is elevated in other fibrotic diseases and because conceptually HERV-K could promote fibrogenesis by inducing cellular stress. Moreover, HERV-K expression is reportedly enhanced in response to herpes virus infection. HERV-K protein NP9 can negatively regulate EBV EBNA2 expression by binding to EBNA2 [43]. The env-encoded superantigens SAg and NP9 were increased in EBV-transformed lymphocytes, and further studies have demonstrated that the EBV genes LMP2A and LMP1 transactivate HERV-K gene expression [44, 45]. Given the reported association between EBV and HERV-K, we hypothesized that gene expression of EBV and HERV-K should have a positive correlation. As such, we performed RT-PCR for HERV-K and EBV, and found expression of HERV-K env and LTR, but no or low expression of EBV (Fig. 2 and Fig. 1a). The absence of differences in overall HERV-K expression between IPF and control data further supports the concept that there is no association between EBV and IPF.

Although RNA-seq is highly sensitive technique to detect virus, there are still some limitations in our study. Most of the mapped viral reads detected in our study were low except for HERVs, and this potentially could be due to the quality of RNA-seq or the script used in our research. Additionally, the expression of RNA viruses is difficult to differentiate from genome detection despite the fact that viral RNA may indeed reveal expression of DNA viruses such as herpes. For persistent DNA viruses with very limited expression such as HBV, a strategy restricted to the detection of RNA such as RNA-seq, may miss these viruses. Finally, although a greater number of tissue samples would add to the confidence of our findings, we suggest the lack of significant findings in 28 IPF lungs from 3 different sources is compelling. Although our sample size makes it very unlikely to have missed a significant difference in expression of viral gene expression in common viruses, such as EBV, we are not able to fully exclude that there may be a small percentage of patients with IPF, or a specific IPF phenotype, that have expression viral RNA in lung.

This study has bearing as clinical investigators are considering anti-herpesvirus therapy as a treatment for IPF. A similar scenario existed for glioblastoma multiforme (GBM) in which, based on IHC, in situ hybridization, western blotting and RT-PCR, CMV was entertained as a causative factor for this fatal disease [46]. A clinical trial using anti-viral therapy to treat GBM was not effective [47]. Only after conclusion of the trial did next generation sequencing data come to light that refuted the role of CMV in GBM [48]. Our data indicates that a clinical trial employing anti-herpesvirus medication for the treatment of IPF would be unwarranted, with the caveat that it does not address so called acute exacerbations of IPF.

## Conclusions

Our study employs next generation RNA-sequencing to assess whether viral infections are linked to the pathogenesis of IPF for the first time. Although quantification of viral RNAs using RNA-seq in IPF lung specimens does not support the role of viral infection in acute exacerbations of IPF, however, this analysis patently did not support an association between virus detection especially herpes virus detection and IPF.

## Supplementary information

**Additional file 1: Table S1.** Counts for human and virus mapped RNA-seq reads for IPF and control lung RNA. Virus data is shown if at least one read was detected in at least one sample.

**Additional file 2: Table S2.** Counts for human and HERV-K & HERV-W mapped RNA-seq reads for IPF and control lung RNA. Virus-encoded genes are shown if at least one read was detected in at least one sample.

**Additional file 3: Table S3.** Counts for virus mapped non-poly(A) selected RNA-seq reads for IPF and control lung RNA.

**Additional file 4: Table S4.** Counts for HERV mapped non-poly(A) selected RNA-seq reads for IPF and control lung RNA.

**Additional file 5: Table S5.** Counts for virus mapped SRA RNA-seq reads for IPF and control lung RNA.

## Data Availability

All data generated or analyzed during this study are included in this published article and its supplementary information files. Raw and processed RNA-seq data for the first group is available at Gene Expression Omnibus [49], accession numbers: GSE138239 for poly(A) selected RNA-seq data and GSE138283 for non-poly(A) selected RNA-seq data. The second and third group RNA-seq data have been previously uploaded to GEO and the lung genomics research consortium (www.lung-genomics.org) as shown in their publications [22, 23].

## Abbreviations

CCL12: Chemokine (C-C motif) ligand 12
CCL2: Chemokine (C-C motif) ligand 2
CMV: Cytomegalovirus
EBER: Epstein–Barr virus-encoded small RNA
EBNA: Epstein-Barr nuclear antigen
EBV: Epstein–Barr virus
ELISA: Enzyme-linked immunosorbent assay
EMT: Epithelial-mesenchymal transition
env: Viral envelope
ER: Endoplasmic reticulum
FISH: Fluorescence in situ hybridization
GAPDH: Glyceraldehyde 3-phosphate dehydrogenase
GBM: Glioblastoma multiforme
HCMV: Human cytomegalovirus *or human betaherpesvirus 5*
HCV: Hepatitis C
HERV-E: Human endogenous retrovirus E
HERV-K: Human endogenous retrovirus K
HHV-2: Herpesvirus 2
HHV-6, − 7 and − 8: Human herpesvirus-6, − 7 and − 8
HHV-6B: Human betaherpesvirus 6B
HHVs: Human herpes viruses
HSV-1: Herpes simplex virus type 1
IFN-γ: Interferon gamma
IHC: Immunohistochemistry
IPF: Idiopathic pulmonary fibrosis
KSHV: Kaposi’s sarcoma-associated herpesvirus
LMP1: Epstein-Barr latent membrane protein 1
LTR: Long terminal repeat
LTRC: Lung tissue research consortium
ORF: Open reading frame
PBMC: Peripheral blood mononuclear cell
PERK: Protein kinase R (PKR)-like endoplasmic reticulum kinase
RPHM: Reads per million human mapped reads
RT-PCR: Reverse transcription polymerase chain reaction
RT-qPCR: Quantitative reverse transcription PCR
SRA: Sequence read archive
TGF-β: Transforming growth factor beta
TNF-α: Tumor necrosis factor alpha
TTV: Transfusion transmitted virus
UPR: Unfolded protein response
UTR: Untranslated region
VCA: Viral capsid antigen
